# *“Even if the test result is negative, they should be able to tell us what is wrong with us”*: a qualitative study of patient expectations of rapid diagnostic tests for malaria

**DOI:** 10.1186/1475-2875-12-258

**Published:** 2013-07-22

**Authors:** Evelyn K Ansah, Joanna Reynolds, Samson Akanpigbiam, Christopher JM Whitty, Clare IR Chandler

**Affiliations:** 1 Dangme West District Health Directorate, Ghana Health Service, PO Box DD1, Dodowa, Ghana; 2 Department of Global Health & Development, London School of Hygiene & Tropical Medicine, 15-17 Tavistock Place, London WC1H 9SH, UK; 3 Department of Clinical Research, London School of Hygiene & Tropical Medicine, Keppel Street, London WC1E 7HT, UK

**Keywords:** Rapid diagnostic tests, Diagnosis, Malaria, Patient perceptions, Expectations, Test-driven, ACT, Targeting

## Abstract

**Background:**

The debate on rapid diagnostic tests (RDTs) for malaria has begun to shift from whether RDTs should be used, to how and under what circumstances their use can be optimized. This has increased the need for a better understanding of the complexities surrounding the role of RDTs in appropriate treatment of fever. Studies have focused on clinician practices, but few have sought to understand patient perspectives, beyond notions of acceptability.

**Methods:**

This qualitative study aimed to explore patient and caregiver perceptions and experiences of RDTs following a trial to assess the introduction of the tests into routine clinical care at four health facilities in one district in Ghana. Six focus group discussions and one in-depth interview were carried out with those who had received an RDT with a negative test result.

**Results:**

Patients had high expectations of RDTs. They welcomed the tests as aiding clinical diagnoses and as tools that could communicate their problem better than they could, verbally. However, respondents also believed the tests could identify any cause of illness, beyond malaria. Experiences of patients suggested that RDTs were adopted into an existing system where patients are both physically and intellectually removed from diagnostic processes and where clinicians retain authority that supersedes tests and their results. In this situation, patients did not feel able to articulate a demand for test-driven diagnosis.

**Conclusions:**

Improvements in communication between the health worker and patient, particularly to explain the capabilities of the test and management of RDT negative cases, may both manage patient expectations and promote patient demand for test-driven diagnoses.

## Background

The introduction of more effective but more expensive artemisinin-combination therapy (ACT) for malaria has been accompanied by continued mis- and over-diagnosis of malaria, and over-prescription of ACT. Reflecting efforts to maximize the cost-effectiveness of ACT, reduce the chance of resistance, and increase the number of non-malarial febrile illness cases correctly treated, the World Health Organization (WHO) now recommends prompt parasitological confirmation by rapid diagnostic tests (RDTs) or microscopy for all patients suspected of malaria before treatment is started [1]. The scale-up of RDTs has been rapid [2] but the use of tests has not always translated into rational use of anti-malarials as a considerable proportion of test-negative patients have been found to receive anti-malarials in some settings [3-10].

Though the introduction of RDTs may result in increased access to parasitological testing for malaria, particularly in low-resource settings where availability and quality of microscopy may be lacking [5], the technological potential and cost-effectiveness of RDTs are compromised if providers do not always follow test results in their prescribing behaviour [11]. Studies of RDT introduction have involved various deliberate or incidental interventions, commonly training and supervision of health workers. These studies have shown mixed results, with some showing a significant decrease in prescription of ACT following a negative test result (see for example [12-14])and others showing continuing high levels of over-prescription of ACT (see for example [4,7,8]). Different levels of adherence to results has in part been attributed to the need for closer alignment of supporting interventions with health worker needs in the context of local cultures of clinical care [12,15-17]. All consultations are, however, a complex interaction between patient and clinician, and the influence of the patient on a clinician’s practice has been recognised [18,19]. This suggests a need to look beyond the providers and to consider the role patients may play in the appropriate diagnosis and treatment of malaria.

There is limited literature examining patients’ perceptions of RDTs. In general, these studies have explored the ‘acceptability’ of the use of RDTs to communities, including patients and caregivers of children [20-25], often reporting high levels of acceptability and positive perceptions of RDTs. However, it is important to understand not only whether tests are acceptable or not to those receiving them, but also how the tests are experienced by patients in practice; studying the technology *in action*, from the patient’s perspective [26]. This can help to identify the network of relations and practices that may shape how a RDT is interpreted and understood by patients, providing an opportunity to explore mechanisms through which the role of the patient may influence the process of diagnosis and treatment for malaria and following a negative test result. To this aim, a qualitative study was conducted to explore the experiences with RDTs of patients and caregivers who had received a negative test result within the context of a randomized controlled trial in public health facilities in Ghana [8].

## Methods

### Study context

The study was carried out in the Dangme West District in southern Ghana, a purely rural district with an estimated 2009 mid-year population of about 142,633. The population lives in scattered small communities of less than 2,000 people. The district is divided into four administrative subdistricts. There are a total of 17 health facilities serving the population. These include four health centres and six community-based clinics. Private sector facilities include three private clinics and two private maternity homes. There is one publicly owned laboratory in one large health centre at Dodowa and two privately owned laboratories in two other subdistricts. There is also a mission clinic as well as a quasi-government clinic. The doctor: population ratio at the time of the study was 1: 23,722. The district had no hospital at the time of the study. One of the health centres was in the process of being upgraded to a district hospital status at the time of the study.

Malaria accounted for about 50% of all reported cases at the outpatients department in all health facilities and most of these cases were as a result of presumptive diagnosis without parasitological confirmation. In the health centre where laboratory facilities did exist, on average, 68% of malaria diagnosis was confirmed by a laboratory test at the time of the study.

At the time of the study, there had been a policy change from chloroquine as a first line antimalarial to artesunate amodiaquine. However, some non-literate community members still tended to call any antimalarial “chloroquine”. The antimalarials prescribed for patients diagnosed with malaria were artesunate amodiaquine or artemether lumefantrine, the latter being the alternate first line antimalarial. Chloroquine was no longer being dispensed at health facilities at the time of the study and the trial documented all drug prescriptions in the patients’ folders.

### Study design

This study formed one part of a qualitative evaluation of a randomized controlled trial to test the impact of RDTs on prescription of anti-malarials in health facilities with microscopy or presumptive treatment as standard care [27]. The quantitative results of the trial indicated that the introduction of RDTs in facilities with existing microscopy had little impact on prescribing behaviour, but in facilities previously only using presumptive treatment there was a significant reduction in over-prescription of anti-malarials. The other part of the qualitative evaluation of this trial explored clinicians’ experiences with using RDTs in the trial [16]. The findings indicated that meanings of RDTs were constructed variously by clinicians through interactions with colleagues and patients, and through different forms of participation with the tests themselves, resulting in changed practice for some clinicians but reinforcing existing practice in others.

This qualitative study was undertaken at four of the health facilities included in the trial in Dangme West District, of which, one, health facility C, had facilities for microscopy and three primarily practised presumptive malaria diagnosis and treatment. The health facilities in the study varied in size and composition: health facility C was a large health centre with a high patient load where one medical doctor and four medical assistants provided curative care, supported by 10 nurses. Health facilities A and B were smaller. One was a small health centre (B) whilst the other was a community clinic (A). Both had five prescribers, including one medical assistant at the health centre. Health facility D was a private clinic with one medical assistant prescribing.

Patients who had recently been tested with an RDT with a negative test result in the study health facilities were eligible for participation in focus group discussions (FGDs), as were mothers of children less than five years of age whose children had tested negative with an RDT. Recruiting only those who had received a negative test result reflected interest in the practice of over prescription of anti-malarials for patients without a confirmed malaria diagnosis. To explore this in more depth, the sample was stratified by two subcategories: 1) those who had been prescribed anti-malarials following their negative test, and 2) those who had *not* been prescribed anti-malarials. FGDs were also segregated by gender to allow for more open discussion (see Table 1 for a breakdown of the FGDs by subgroup). Two FGDs were planned for patients recruited to the trial at each of the larger health facilities (A and C); only one FGD was planned to represent each of the smaller facilities where recruitment was slower and could have affected recall for patients enrolled a long time before the FGD.

**Table 1:** Description of selection criteria of clients participating in the FGDs

**Focus Group**	**1**	**2**	**3**	**4**	**5**	**6**
**Number of FGD participants**	9	8	9	10	8 Plus 1 IDI	9
**Health facility**	A	A	B	C	C	D
**Patient type**	Female	Male	Female	Male	Mothers	Mother
**RDT result**	Negative	Negative	Negative	Negative	Negative	Negative
**Anti-malarial (AM) prescribed or not**	AM prescribed	AM prescribed	No AM prescribed	No AM prescribed	AM prescribed	No AM prescribed

Sampling for the FGDs was systematic. After seeking permission from community leaders to conduct the FGDs, potential respondents who represented the sub-groups of interest were identified from the records of the main trial about six weeks after the trial ended. Those recruited into the trial most recently were invited first to participate, and further participants were identified and invited by working backwards in time through the trial database. No more than five people were selected from any one day of the trial, in order to reflect a variety of clinician and day circumstances at any one facility. Twelve community members were invited for each focus group. If any declined at the time of invitation, the plan was to note them in the study log and select further people using the same selection procedure. However, none of the respondents invited to participate declined to do so.

### Conducting focus group discussions

FGDs with community members were conducted within the communities in which the health facilities were located, and guidance was sought by community leaders as to the most appropriate time and place to hold the discussions. Each potential respondent was contacted by the study team by phone or face-to-face visit to invite them to the group discussion. FGDs were conducted between 20 March and 8 April, 2009.

After giving information about the study and gaining consent from respondents, FGDs were conducted in the local language. Confidentiality and anonymity were assured, ground rules were discussed with each group and permission was sought to record the discussions. A semi-structured topic guide was followed, focusing on perceptions of malaria risk and symptoms, perceptions of and experiences with RDTs, trust in test results, perceptions of treatment, and interaction with clinicians. No incentive was provided, other than refreshments and reasonable transportation costs.

In addition to the audio recordings, demographic data were collected from respondents and notes of the discussion were taken by a note-taker to capture non-verbal communication, and information about the setting and atmosphere. Immediately after each FGD, a contact summary form was completed to capture the discussion content and atmosphere, identify any emerging themes and make any suggestions for revisions to the topic guide. All respondents’ remained anonymous after the discussion using pre-assigned identification numbers.

### Data analysis

Audio files were transcribed phonetically in the original language (Dangme or Ga) and then translated into English. Sections of text were double-checked for accuracy of translation by other members of the field team. Transcripts were then imported into QSR Nvivo 9. Coding took place through an iterative process. Coding was done line by line to generate a ‘node tree’ describing the contents of the data across each transcript. Each transcript was then revisited to re-allocate text to new codes where appropriate. The nodes were then grouped into categories to generate cross-cutting themes. These emerging themes were analysed according to different subgroups of the respondents, by the categories “negative, received an anti-malarial” and “negative, received no anti-malarial” to see if the themes applied to different groups. The themes that emerged from the data were also considered in the light of the initial conceptual framework to generate findings.

### Ethics

The ethical review boards of Ghana Health Service and the London School of Hygiene & Tropical Medicine approved the study. The trial which preceded this study was prospectively registered at ClinicalTrials.govNCT00493922.

## Results

### Respondents

The total number of FGDs conducted was six, with an average of nine respondents in each, and 53 respondents in total (see Table 1). An additional in-depth, one-on-one interview was conducted with a mother from health centre C who arrived late for the FGD and requested to participate. She did not attend the FGD and as such was unaware of the responses the group members had given earlier. The transcript from this interview was coded alongside the corresponding FGD from the same facility.

### Overview of results

Respondents had high expectations of RDTs. They perceived a valuable role for RDTs as part of a process to help the clinician reach the right diagnosis and treatment decision. RDTs were also described as useful for communicating their problem to the clinician. However, narratives of the experience of being tested portrayed conceptualizations of the RDT as a generic test able to identify *any* cause of illness, not just malaria, and expectations that a test should result in a diagnosis, even following a negative result. Respondents also identified a limited effort by clinicians to engage patients in the process by which their illness was diagnosed and treatment prescribed. Clinicians appeared to retain strong hierarchical distinctions, played out in dismissive attitudes and lack of communication. As such, the tests appeared to have been adopted into an existing opaque system, with hidden processes of tests and silent processes of diagnostic and treatment decisions.

Although there was a small amount of variation in responses within and across focus groups, this did not seem to be related to the different subgroups of respondents (males, females and caregivers) or to whether or not they had received an anti-malarial following their negative test result. In the discussions with caregivers (mothers), many respondents talked about their personal experiences of being tested, as well as experiences of their children, and often the two were not easily distinguishable. The themes and constructs presented below were represented in the different sub-groups and FGDs (except where otherwise indicated), and the quotations have been selected to be illustrative of these key themes, rather than reflect each FGD.

### Understandings of malaria

To contextualize the findings, it is important to present respondents’ understandings of malaria and its symptoms. In most discussions, malarial illness was attributed to a range of factors, including the weather, poor hygiene, working hard and mosquitoes. These do not directly relate to the concept of malaria parasites being the direct, and only, cause of ‘true’ malarial disease. Respondents were very familiar with biomedical signs and symptoms of malaria, such as fever, body pain and weakness. Some signs and symptoms of complicated malaria were described in detail:

*“Sometimes you can begin to see things and you can leave the house and go to places you are not conscious of”.* (Respondent 04; FGD4)

In general, many respondents perceived malaria to be a “*bad illness”*.

*“Malaria is a bad illness. It is capable of rendering the body weak. It can reduce your strength or affect your level of intelligence. That is how I see it”.* (Respondent 06; FGD 2)

*“When the child has high temperature and you keep the child in the house, his condition would worsen and you would realize that the child will become very weak. His eyes also changes and if you don’t rush the child to the clinic it can kill him”.* (Respondent 02; FGD 6)

### Finding a cure: high expectations of rapid diagnostic tests

#### RDTs improve clinical decisions

When asked about their experiences with the ‘new test’, and/or the ‘RDT test for malaria, respondents described the test as a valuable addition to the clinician’s process of diagnosis and treatment of their illness,

*“Doctors are not soothsayers who can just look at your face know what is wrong with you. He has to test you”.* (Respondent 06; FGD 2)

RDTs were seen as contributing to the clinician’s diagnostic and treatment decision by revealing the cause of illness present in the patient’s blood,

*“I also think it is the test that would reveal the exact cause of the illness; that is why you are asked to go for the test. That is what I think”.* (Respondent 05; FGD 3)

The tests were seen to enable the clinician to make an accurate diagnosis that would lead to the prescription of the ‘right’ treatment, which would *“cure the disease”* (Respondent 06; FGD 4). A number of respondents indicated that the treatment prescribed following a test would be more effective than drugs taken without testing, resulting in a quicker recovery and removing the need to seek care multiple times for the same illness:

*“When you are tested, the problem is solved once*”. (Respondent 06; FGD 6)

*“It is the test that can help us. If we don’t do the test and the Doctor gives us drugs, the sickness may not stop because it may be due to other ailments and not malaria alone. That is why the test is required”.* (Respondent 01, FGD 4)

### The test can identify any cause of sickness

Respondents went beyond the idea that RDTs were able to detect malaria specifically, which was described by some, to convey an understanding that the test could detect in the blood any cause of illness. As such, the test was perceived as important for enabling the doctor to “*properly diagnose the disease”* (Respondent 03, FGD 5) whatever that may be. Consequently, the respondents expected that if the test produced a negative result for malaria, it should reveal an alternative cause for their illness, to guide the clinician’s prescription of treatment:

*“Even if the test result is negative, they [*the clinician*] should be able to tell us what is wrong with us or tell you that it is not malaria but rather this sickness and prescribe drugs for us”.* (Respondent 05; FGD 1)

Respondent stories showed how the RDT was seen as a central activity in finding the cause of the illness.

*“When you are tested, they can diagnose the cause of your illness*”. (Respondent 03; FGD 2)

*“I think because you don’t know what the cause of your illness is, that is why they ask you to go for the test so that they will know what the cause is and give you medication. This is my view”* (Respondent 03; FGD 3)

The test was thought to be able to tell at what stage the malaria was, in order to prescribe the appropriate dose of medication to deal with it as indicated in this example below:

“*Even if the doctor knows you have malaria he would like to conduct the test to determine the stage of the disease so that he can give you the right dose of the drugs that can cure the illness”. *(Respondent 09; FGD 5)

### The test can communicate the problem to the doctor

Several discussions suggested the test was perceived to play an important role in communicating on behalf of the patient to the clinician. Respondents explained that sometimes they and patients in general, were unable to express themselves well or explain their symptoms clearly to the clinician. The test provided a means for clinicians to get around this gap in communication, as it was perceived to convey better their signs of illness:

*“Sometimes we don’t explain ourselves well to the doctors, so they will ask us to go for the test to be sure of what the problem is before they give us drugs”.* (Respondent 04; FGD 4)

These perceptions of the inadequacy of patients’ communication of symptoms, in comparison with the test, were framed by broader descriptions of the challenges of interacting with clinicians. These arose from more generalized statements about experiences of clinicians’ negative attitudes towards patients, not necessarily linked to their experiences of testing:

*“Sometimes when you start discussing your problem, then they begin to shout at you. The next time you go to the clinic, you will find it difficult to tell them the truth or discuss your problem with them because of what happened the previous visit”*. (Respondent 01; FGD 6)

Thus, one of the ways that RDT enabled the right diagnosis and treatment for a patient was through communicating their problem in a way that was acceptable to clinicians.

Although other research on RDTs has indicated that some people confuse the malaria test with tests for HIV, and fear the possibility of it revealing a positive HIV status (for example see [21]), only one respondent in this study articulated a question of whether the RDT could detect HIV. Rather, the RDT was widely conceived as an all-purpose means by which clinicians would be able to identify the cause of illness.

### Hidden processes and silent diagnoses: limited patient engagement

#### Testing as a hidden process

When asked to describe their experiences with procedures of testing with RDTs, respondent accounts lacked details of visual or technical aspects of the tests and focused on experiences of having blood taken and waiting time.

“*They took my blood and asked me to wait. When he brought the result he said he did not find anything in the blood and I was told to go*”. (Respondent 04; FGD 2)

Comparisons were made with microscopy in this regard. Some thought the quantity of blood taken for the new test was less than for microscopy, and as a result the test was less painful which was preferable. In addition, many respondents were positive about the RDT taking less time than microscopy at the laboratory, and reported not minding waiting for the RDT result to be produced. Several respondents suggested that this waiting time was an important feature of the process through which the clinician would determine the cause of illness via use of the test:

*“Because your aim is to get well the time is not an issue. You don’t know what the doctor has seen that he has asked you to go for the test. You must always exercise patience so that the doctor can know what is wrong with you and give you medication. If you really want to be cured you should have patience. The time should not be an issue”.* (Respondent 01; FGD 3)

The lack of detail of the test itself may reflect the separation of patients from laboratory procedures. The trial introduction of RDTs followed standard practice in Ghana to perform tests in a laboratory or special room, separate from patients and from the consulting room, and to return the results to the clinician to interpret.

When describing testing procedures, respondents often referred to RDTs as a *“machine”,* using the English word, despite communicating in the local Ga-adangme language. This identifies the test as a technology outside of the local or lay domain, conceived as complex and not clearly understood, and occurring separately from the usual consultation with the clinician. This conceptualization indicates a perceived mystification of the technological process of the test, which may contribute to the expectations held by respondents of the test’s capacity to diagnose any disease.

*“I don’t understand the new test. When they take your blood sample, which machine do they put the blood sample in? What do they do with it?”* (IDI)

### Results unknown: part of a silent diagnosis

Patients gave mixed reports of whether they were told the results of the test or not. Laboratory staff, in the main, left the communication of test results to the clinicians. Some patients reported not having received the results of RDTs at all or not directly during the consultation with the clinician, exemplified in this group discussion:

***“Moderator (M):****Was the result of the test disclosed to you?*

***07:****They never told me what was wrong with me.*

***04:****Even though the test result shows I had no malaria, because of the way I was feeling, the Doctor said they should give me some malaria drugs.*

***05:****I was not told the result of the test. He [*the laboratory technician*] asked me to take the result to the nurse [*clinician*] and it was the nurse [*clinician*] who told me I had malaria.*

***02:****No, they asked me to see [*name of nurse*] and when I went the nurse [*clinician*] told me.*

***01:****No, they did not tell me anything, not even the nurse [*clinician*]”. *(FGD 2)

In many cases, the hidden nature of the testing process appeared to be extended to the results themselves, incorporated into an opaque diagnostic process by the clinician:

*“The issue is that the result of the test is stapled and so you cannot open it. It is the Doctor who opens it and he can decide to tell you or not”. *(Respondent 06; FGD 4)

“The lab technician told us that he will send the result to the Doctor when he comes. He told me he does not know the result of the test but will show it to the Doctor when he comes”. (Respondent 06, FGD 2)

Responses of some patients suggest they interpreted a malaria diagnosis even though, according to trial records, their RDT was negative. This could reflect a misinterpretation of interaction with the clinician or, for those who were prescribed anti-malarials following a negative test, an interpretation of receiving this prescription as indicative that they had been given a malaria diagnosis. It is also possible that respondents were reflecting on different treatment seeking episodes than the case recorded in the trial, limiting our ability to explore this theme in more detail in our data. However, if malaria diagnoses were inferred for RDT negative cases, this suggests further potential consequences of the silent process of diagnosis described by respondents.

### RDTs and the locus of knowing: clinicians cannot be questioned

Many respondents conveyed their unwillingness to question or challenge the clinician’s decisions around whether to test and how to respond to the test result.

*“I am always glad when the Doctor asks me to go for lab test because I would be wondering what is causing my illness. Sometimes I feel like telling the Doctor I want to go for lab test but I don’t know what is on his mind. So when he eventually tells me to go for the test I become happy”.* (Respondent 08; FGD 3)

Descriptions of clinicians as *“second gods”* conveyed perceptions of the level of authority held by them, and this underpinned respondents’ statements about the clinician alone knowing how to interpret a test and prescribe the right treatment:

***05****: “You are talking about a situation where I am expecting that the Doctor will give me a specific drug but he gives me a different drug.*

***M:****Yes*

***05:****Doctors are our second gods. Whatever sickness they will find in your body they will give you drugs that will cure that disease. So I will not be bothered”.*

(FGD 1)

*“When you are sick and you go to the clinic and you go for the test and the test reveals the cause of your sickness, it is only the doctor who can find solution to that problem. So it all depends on what the doctor thinks is the right thing to be done”.* (Respondent 04; FGD 4)

Many respondents conveyed their role as a passive recipient of care, entrusting the clinician -with the responsibility to know and prescribe what was ‘right’ for them. The enactment of this role, coupled with expectations of clinical authority, meant clinical decisions should not be questioned by the patient:

*“We will accept any drug the Doctor will give us. He has conducted test on us and has seen the result, so whatever drug he will gives us we can’t say anything. This is what I think”.* (Respondent 01; FGD 3)

*“We cannot say anything. We can’t tell the Doctor to give us paracetamol, chloroquine, blood tonic or malaria drugs. It is what we tell them and what they will diagnose that will inform them about the kind of drug he will give us”. *(Respondent 01; FGD 1)

This theme of acceptance of the clinician’s prescribed treatment emerged alongside some respondents’ statements that they were not told the results of the RDT, and therefore, presumably, were not informed of a test-based diagnosis of their illness. This stands in contrast with the expressed desire and expectation by many respondents, based on their understanding the RDT, that they should be told the exact cause of their illness after testing.

### Situations of patient action

In contrast with the lack of involvement in testing, diagnostic processes and decisions, some patients felt they could play a role in some decisions. Within the clinical encounter, it appeared acceptable to negotiate regarding the specific medicine prescribed once a diagnosis has been made, for example to inform the clinician if the drug he prescribed had caused unpleasant effects in previous experiences and to request something else:

*“Chloroquine is good for some people but chloroquine is not good for me. Even if I take four tablets, my body will itch for about a week. What I normally do is I tell the Doctor what chloroquine does for me and he will either change it or add some drugs to stop the itching”.* (Respondent 08; FGD 3)

Patients also described their ability to find alternative solution to their illness outside of the health facility environment. While they would not challenge the clinician’s prescription, they would seek care elsewhere if dissatisfied with the drug treatment or if perceiving it not to work. Community drug stores were mentioned in several discussions as alternative sources of care:

*“If the drug doesn’t work, you can go to the drug store and explain your condition and see if you can get other drugs”.* (Respondent 03; FGD 1)

*“I went for the lab test on Friday but I bought some drugs over the weekend from the drug store (Maladrin). On Monday I went back to see the Doctor with the lab result and I showed him the drug I took”.* (Respondent 01; FGD 4)

## Discussion

The debate on RDTs has begun to shift from whether RDTs should be used, to how and under what circumstances their use can be optimized [1]. Calls for the increased availability of RDTs in both the public and private sectors, as part of efforts to rationalize the use of ACT [28,29] have increased demand for a better understanding of the complexities surrounding the role of RDTs in appropriate treatment of fever. This qualitative study has added to, existing understanding of clinicians’ interaction with RDTs following their introduction to health facilities in one district in Ghana [8,16], to explore the perceptions and experiences of patients and caregivers of children who had been tested as part of the trial.

In this study, patients who had been tested with RDTs held positive, but often technically inaccurate, perceptions of RDTs, and considered their role in accurate diagnosis and treatment of their illness to be valuable. Their expectations for the RDT to identify any cause of illness – not just malaria – could however in time compromise this apparent acceptability of the tests as they become more established in the health care context if not addressed, as it cannot be realized. Patient experiences of RDTs were embedded in existing hierarchical social relations between clinicians and patients, with patients perceiving limited ability to engage in the clinical process and to influence providers’ behaviour around testing and treating malaria. The finding that the technical mechanisms of RDTs were not seen to be part of the lay domain, combined with the perception of the diagnostic process as housed in the clinical domain, suggests that patient demand for treatment to directly follow test results in this setting is currently limited.

The highly positive perceptions of RDTs demonstrated by patients in this study correspond closely with other similar studies conducted in Ghana, Tanzania and Uganda [20,21,23-25]. Others have also identified that RDT acceptability is based on its perceived role in aiding the clinician to reach a more definitive diagnosis and prescribe the right treatment for their illness [20,23]. The value of the test in communicating a patient’s ‘problem’ to the clinician more clearly than they could verbally, has also been reported elsewhere [24]. However, beyond this, it was found that perceptions of RDTs were underpinned by unrealistically high expectations from the tests. Respondents in the study perceived the RDT as able to detect any cause of illness, beyond malaria. This may indicate interpretations of the technology as an ‘object of hope’ in terms of curing illness [30] and may reflect broader ideas that the quality of health care is equated with use of new technologies like RDTs [23], framed by an assumption that problems are resolvable through technological means [31].

Study respondents described the test as a ‘*machine’,* a descriptor also reported for RDTs elsewhere in Ghana [24], suggesting recognition of a highly technological and complex process by which a result is produced. Beyond this, patients were unable to describe the processes of the RDT. Rather, the position of testing in a laboratory or special testing room outside of the lay purview, seemed to allocate to the test a status of mystery and power, described for biomedical investigations elsewhere [32]. This finding contrasts with the other study of patient experiences with RDTs in Ghana where patients were able to describe clearly the way the tests give results, in a context where the patients had attended study clinics, run by well-trained fieldworkers as part of a clinical research team [24]. It is possible that there the test was carried out more openly by the fieldworkers, along with explanations about the tests to patients. This may have affected some of their perspectives of the test as a more objective indicator of malaria than clinical opinion. In this study, where testing was incorporated into routine services, patients presented themselves as outside of RDT processes. Test results were elusive, and patients did not identify mechanisms by which the test should determine clinical management decisions. This suggests that the tests were not seen as independent objects, but as part of the clinical process under the expertise of the clinician.

The goal to achieve universal parasitological diagnosis of malaria through improved access to testing rests on the assumption that tests, such as RDTs, provide objective criteria for malaria diagnoses. As such, the tests are held to be the locus of knowledge for making a treatment decision. However, the findings of this study suggest that in a routine scenario in Ghana, RDTs were adopted into a practice where the clinician retains the locus of knowledge, and power, for clinical decisions. Patients expected clinicians to use tests and their results alongside clinical expertise to provide appropriate treatment for their diagnosis. This suggests that RDTs did not undermine clinicians’ position and credibility with patients, reported as a fear of clinicians in Uganda [23], but it also suggests that pressure from patients to use RDTs as a primary determinant of anti-malarial usage may be limited in contexts such as this. The finding echoes results from Tanzania where clinician perceptions of patient pressure for anti-malarials were not upheld by patient expectations for the right diagnosis and treatment [33].

### Implications

The findings presented in this study suggest that, whilst RDTs are clearly welcomed by patients, two issues need to be addressed from the patient perspective in order for RDTs to achieve the potential intended by policy makers and funders. First, expectations that RDTs are able to diagnose any illness need to be managed in order to lessen risks of reduced acceptability of the tests since any mistrust of the new tests among the public will affect their acceptability [34]. Second, and related, the link between RDTs, their results, diagnoses and treatment decisions need to be made transparent. Both can be addressed through improved communication with patients, particularly by health workers themselves. The challenge faced by clinicians is to change an existing system of opaque diagnosis, embedded in a system of clinician authority and patient (blind) trust, to an open encounter that lays bare what is known and unknown in the process of test result-determined decisions. In the analysis of health worker experiences of RDTs in this same trial, respondents conveyed that investing in better communication with patients, particularly in the face of negative RDT results, was difficult at times but worthwhile [16]. Elsewhere, trials to evaluate the impact of the introduction of RDTs alongside supporting interventions that include training in improved communication are on-going and will inform methods for undertaking such interventions. Beyond goals to manage expectations from RDTs and promote test-based diagnoses, improved communication may influence patients’ and caregivers’ understandings of diagnoses and treatment regimens for febrile illness in general, potentially affecting adherence to treatment, perceptions of quality of care, and future care seeking decisions [27,35,36].

### Limitations

The number of FGDs (six) and one interview conducted reflects a fairly small sample size, although no new themes or ideas were identified in the final discussions, suggesting that a level of saturation had been achieved. The study was conducted in a single district, which potentially limits the extent to which the results can be considered applicable outside the specific study context. However, many of the findings correspond with other, similar studies elsewhere including Ghana. The period that had elapsed between the time of visit and the FGD in the case of some respondents may have diminished recall to a certain extent, but this was not evident in the discussions as respondents appeared to talk easily about their experiences. The two groups of caregivers tended to narrate experiences from visits during their own recent illness episode as well as those of their children, and the two were often indistinguishable. It is possible that caregivers had themselves experienced RDTs as part of the trial at another point in time, giving them further insights into the test. In addition, the way testing was discussed, often as ‘testing’ rather than using a word for RDTs, suggests that broader experiences with testing are likely to have been drawn upon in the reflections of respondents. This has the potential to provide a more integrated narrative of RDTs in context but in places it also limited interpretations to testing in general rather than RDTs in particular.

Direct observations of clinician-patient interactions might have offered insight into how patients’ perceptions of RDTs were shaped by the clinical encounter in this context. However, we did not directly observe the interactions between the clinician and the patient as our focus was to understand how patients and local communities made sense of these interactions when RDTs are involved. In addition, direct observation would have had implications for the results of the randomized controlled trial in which this qualitative study was situated.

## Conclusion

When seen as an objective tool to identify malaria cases, RDTs have the potential to contribute to universal parasitological diagnosis and appropriate treatment for all patients suspected to have malaria. When RDTs are adopted into existing scenarios of hidden, or silent, diagnostic processes, where clinicians retain authority that supersedes tests and their results, patients are unable to demand a test-driven diagnosis and this potential is reduced.

The positive perceptions of patients about a diagnostic system incorporating RDTs show these tests as a welcome addition. However, misconceptions of the test’s ability to detect any cause of illness sets patients up for disappointment, particularly if the testing process becomes more transparent. Improvements in communication between the health worker and patient, particularly to explain the capabilities of the test and management of RDT negative cases, may both manage patient expectations and promote patient demand for a more test-driven diagnosis. There is also a need for a corresponding improvement in the communication between laboratory technicians and the patient so that patients can understand what to expect during the testing process and why they need to return to the clinician to discuss the results of the test.

This may increase satisfaction with test-based diagnoses, reduce alternative careseeking and improve acceptance of and adherence to prescribed treatments.

## Abbreviations

ACT: Artemisinin combination therapy; FGD: Focus group discussion; HIV: Human immunodeficiency virus; IDI: Individual in-depth interview; RDT: Rapid diagnostic test for malaria; WHO: World Health Organization

## Competing interests

The authors declare that they have no competing interests.

## Authors’ contributions

EA designed the evaluation, carried out the data collection and analysis and drafted the manuscript. JR contributed to the analysis and drafting of the manuscript. SA contributed to the data collection and drafting of the manuscript. CW contributed to evaluation design and drafting of the manuscript. CC contributed to evaluation design, data collection, interpretation and drafting of the manuscript. All authors read and approved the final manuscript.
